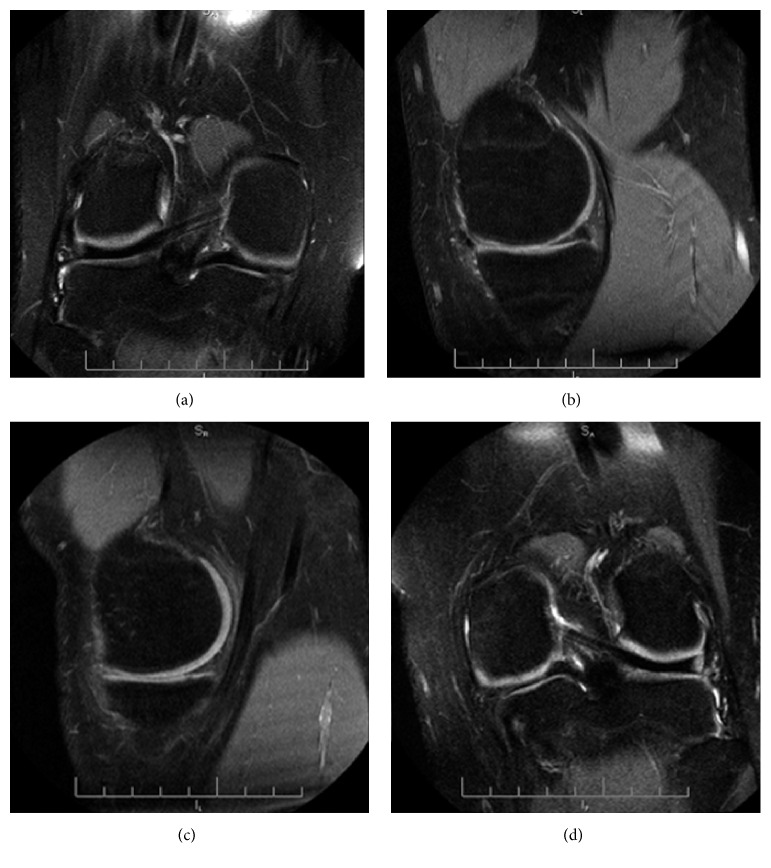# Symptomatic Bilateral Torn Discoid Medial Meniscus Treated with Saucerization and Suture

**DOI:** 10.1155/2016/8487194

**Published:** 2016-08-30

**Authors:** Enrique Sevillano-Perez, Alejandro Espejo-Reina, María Josefa Espejo-Reina

**Affiliations:** ^1^Department of Orthopaedic Surgery, Regional University Hospital of Málaga, 29010 Málaga, Spain; ^2^Hospital Vithas Parque San Antonio, 29016 Málaga, Spain; ^3^Department of Orthopaedic Surgery, Virgen de la Victoria University Hospital, 29010 Málaga, Spain

## Abstract

Discoid meniscus is an anatomical congenital anomaly more often found in the lateral meniscus. A discoid medial meniscus is a very rare anomaly, and even more rare is to diagnose a bilateral discoid medial meniscus although the real prevalence of this situation is unknown because not all the discoid medial menisci are symptomatic and if the contralateral knee is not symptomatic then it is not usually studied. The standard treatment of this kind of pathology is partial meniscectomy. Currently the tendency is to be very conservative so suture and saucerization of a torn discoid meniscus when possible are gaining support. We present the case of a 13-year-old patient who was diagnosed with symptomatic torn bilateral discoid medial meniscus treated by suturing the tear and saucerization. To the best of our knowledge this is the first case reported of bilateral torn discoid medial meniscus treated in this manner in the same patient.

## 1. Introduction

Discoid meniscus is a type of meniscus with an atypical shape, thicker, covering a bigger surface of tibial plateau than a normal meniscus but more fragile which explains the higher frequency of lesions.

The reported incidence rates for discoid lateral meniscus range from 1,2% to 5,2% being the incidence much lower for discoid medial meniscus (0,12–0,3%) [1, 2]. However, in Asian population the reported incidence for discoid menisci ranges from 30% to 50% [3]. There are few reports of medial bilateral discoid menisci in the literature although the real incidence is difficult to determine because an unknown percentage of discoid menisci may be asymptomatic [4].

There are different classifications for discoid meniscus being Watanabe, the most accepted, in which discoid meniscus is classified into three different types according to the arthroscopic aspect: type I or complete, type II or incomplete, and type III or Wrisberg-ligament type in which the posterior meniscofemoral attachment is absent resulting in an unstable meniscus with hypermobility [5]. Jordan classified discoid meniscus depending on its peripheral rim stability as stable type (includes both complete and incomplete types, further divided by the presence of symptoms and tears or not) and unstable type (includes unstable normal and unstable discoid meniscus since both have the same symptoms and treatment) [6].

## 2. Case Presentation

### 2.1. Right Knee

The patient is a 13-year-old male, recreational football player, who presented with pain and is unable to fully extend the knee fully after a low energy impact on his right knee, without episodes of locking or instability.

**Figure 1:**
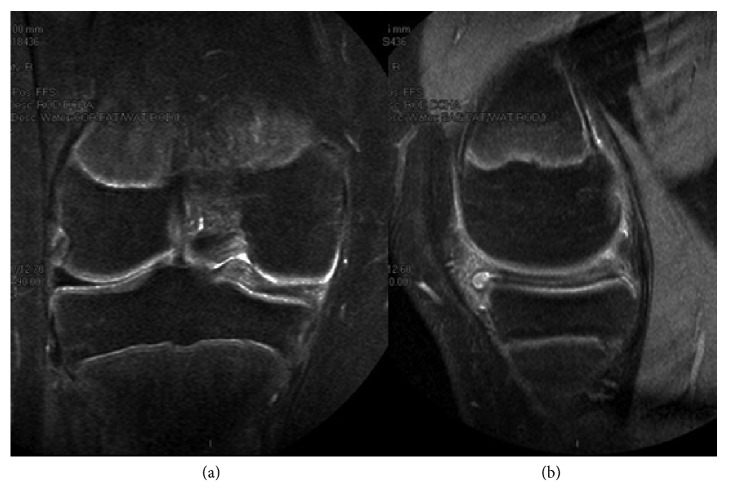
MRI of the right knee. (a) Coronal view showing the upper side of the medial meniscus folded in the intercondylar notch. (b) Sagittal view demonstrating the horizontal tear.

On physical examination the patient had normal alignment of the right lower limb, full flexion with pain on the medial side on the last degrees of flexion and the last 15° degrees of extension; tenderness on the medial joint line, painful click with McMurray test with no effusion, and no ligamentous laxity. Patellar tracking was normal. Simple X-ray of the knee showed no abnormalities and a discoid medial meniscus with peripheral and horizontal tear and the upper side of the meniscus folded in the intercondylar notch was found on magnetic resonance imaging (MRI) scan with no other abnormalities associated (Figure 1).

#### 2.1.1. Surgical Technique

**Figure 2:**
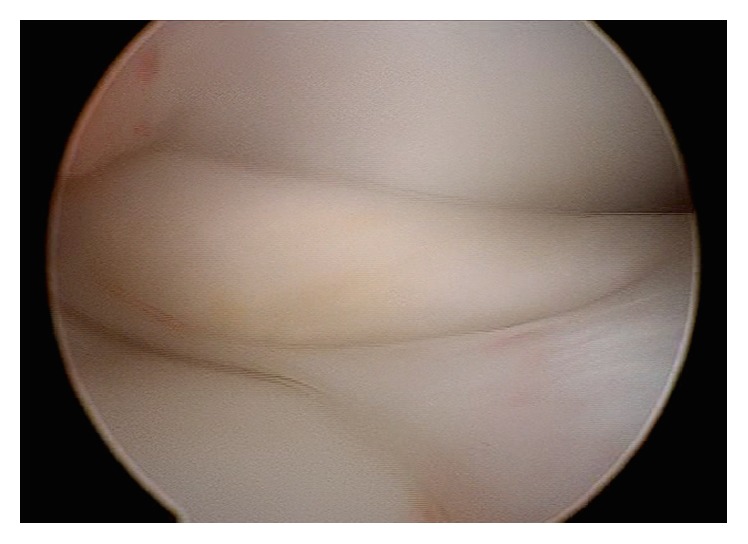
Arthroscopic image of the medial femorotibial compartment of the right knee from the anterolateral portal showing the upper side of the medial meniscus folded in the intercondylar notch.

**Figure 3:**
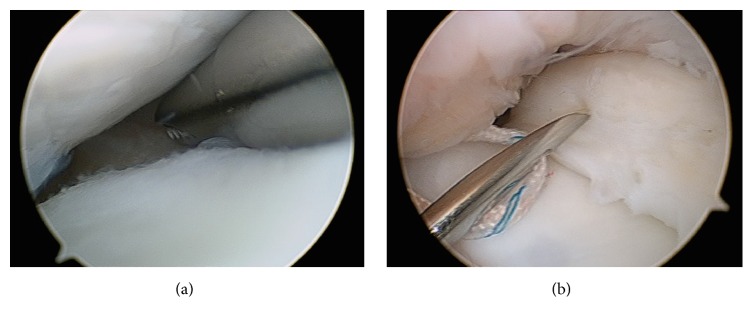
Arthroscopic image of the medial femorotibial compartment of the right knee from the anterolateral portal. (a) Refreshment of the tear with a shaver. (b) Inside-out suture technique of the tear with a specific device.

**Figure 4:**
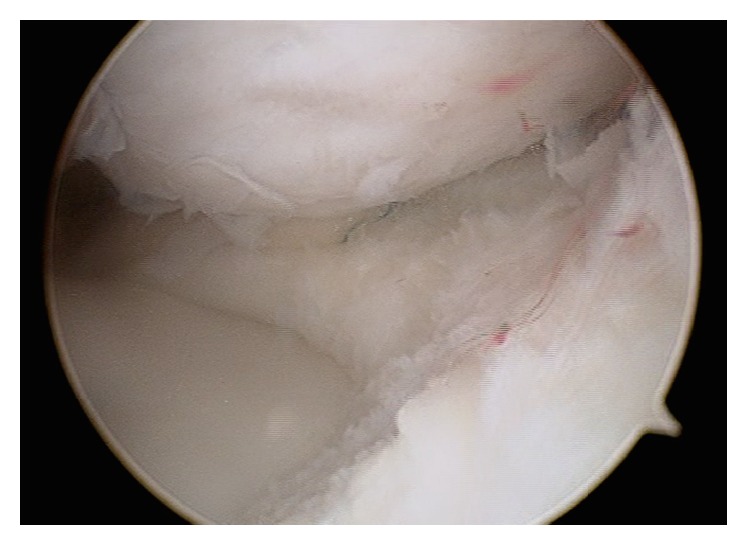
Arthroscopic image of the medial femorotibial compartment of the right knee from the anterolateral portal. Saucerization after the suture.

An arthroscopy of the right knee was performed with the thigh in a leg holder using standard anterolateral and anteromedial portals under general anesthesia. A complete medial discoid meniscus with a partial longitudinal tear in red zone of the body and posterior horn was found. Its upper side was folded to the intercondylar notch behaving as a bucket handle tear (Figure 2). The tear was found to be reducible with a probe. It was refreshed with a shaver (Figure 3(a)) and the meniscus was repaired using an inside-out technique with a specific suturing device (Figure 3(b)) [7] and number 2 Force-Fiber suture (Stryker Endoscopy, San Jose CA). Once the tear was sutured and its stability tested with a probe the body of the meniscus was saucerized with a shaver and radiofrequency at the lowest intensity allowed by the device, to avoid damage to the auricular cartilage, reproducing the shape of a normal meniscus (Figure 4).

Postoperatively the knee was immobilized with a knee orthotic in extension during two weeks and partial weight bearing and limiting flexion to 90° during two more weeks. At three months sport was gradually resumed.

### 2.2. Left Knee

**Figure 5:**
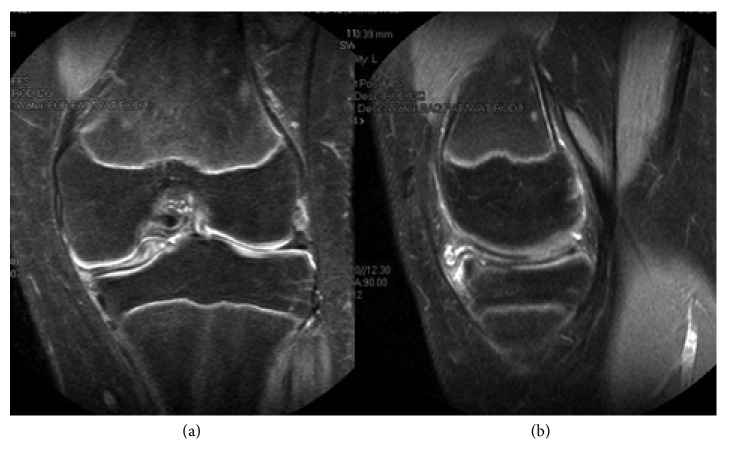
MRI of the left knee. (a) Coronal view showing the upper side of the medial meniscus folded in the intercondylar notch. (b) Sagittal view demonstrating horizontal and vertical peripheral tear.

Six months after the surgery the patient started with pain and incapacity to fully extend the left knee with no trauma associated (and asymptomatic right knee). On physical examination the patient had complete flexion and pain on the last 10° of extension, with tenderness on the medial joint line with no effusion or ligamentous laxity. Patellar tracking was normal. No abnormalities were found on X-ray and MRI showed a tear very similar to the contralateral knee (Figure 5).

#### 2.2.1. Surgical Technique

**Figure 6:**
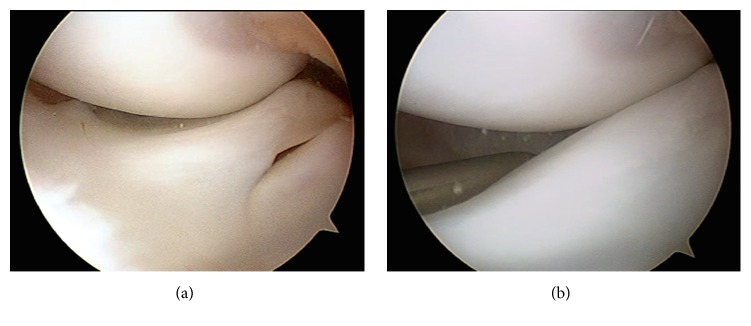
Arthroscopic image of the medial femorotibial compartment of the left knee from the anterolateral portal. (a) A medial discoid meniscus is shown. (b) The upper side of the meniscus is easily displaced into the intercondylar notch with a probe.

**Figure 7:**
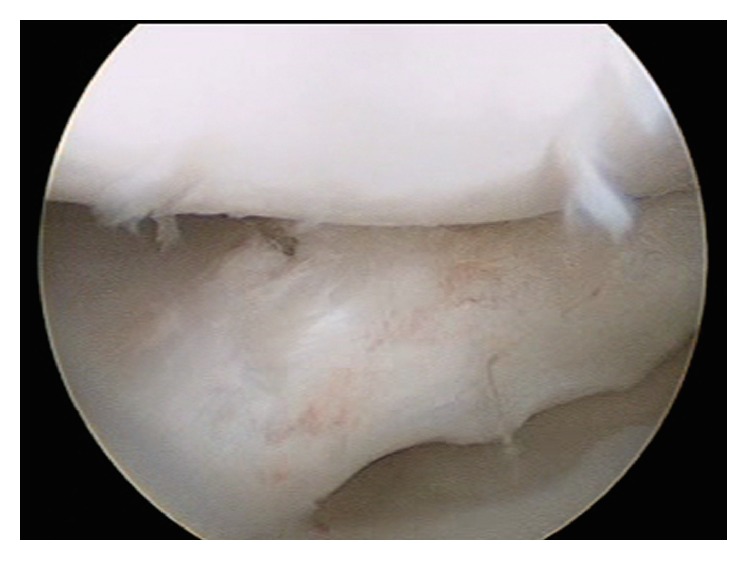
Arthroscopic image of the medial femorotibial compartment of the left knee from the anterolateral portal. Medial discoid meniscus shown after saucerization and suture.

An arthroscopy was performed on the left knee in the same manner as the right one. A complete medial discoid meniscus was found with a longitudinal tear in red zone affecting the body and posterior horn, very similar to the right knee except the upper part of the tear was not folded on the intercondylar notch although it was easily displaced to the notch with the probe (Figure 6). The meniscus was sutured with an inside-out technique using the same specific device and suture used on the right knee. Once the stability of the suture was tested the body of the meniscus was saucerized using a technique similar to that described above (Figure 7). Postoperative care was the same as the right knee.

The patient was reviewed at 6 months, one year, and two years after surgery being asymptomatic and with same preinjury activity level.

MRI was performed two years after the surgery and a reduction of the size and intensity in T2 signal of both repaired menisci was found (Figure 8).

## 3. Discussion

To the best of our knowledge this is the first case published of bilateral medial discoid menisci tear treated with arthroscopic repair and saucerization.

There are different reports of bilateral discoid menisci [8] being very scarce the ones about bilateral medial discoid meniscus [2, 9].

The symptoms of a torn discoid meniscus are usually the same of those caused by a tear of a normal one. In the lateral discoid menisci common finding of “snapping knee” syndrome may help in the diagnosis whereas in the medial menisci it is less specific [10]. In our case the main complaint was knee extension limitation.

Medial discoid meniscus may be asymptomatic and found incidentally after MRI requested for other reasons. In plain radiographs, some abnormalities such as widening of the medial joint margin and cupping of the medial tibia plateau or proximal medial physeal collapse can be found associated with discoid medial meniscus [11]. There are other several abnormalities associated with medial discoid meniscus found on MRI including anomalous insertion of the anterior horn of the medial meniscus into the anterior cruciate ligament, discoid lateral meniscus in the same knee, pathologic medial patella plica, or meniscal cyst [12]. None of these were present in our patient.

The treatment of symptomatic torn discoid meniscus has classically been meniscectomy. Total meniscectomy increases the probability of osteoarthritis compared with partial meniscectomy with a stable peripheral rim [13–15]. Management of torn discoid meniscus has evolved to more conservative surgery. Partial meniscectomy using the saucerization technique resecting the central portion of the meniscus in order to recreate the shape of a normal meniscus has obtained better results in medial and long-term follow-up for torn discoid meniscus than total meniscectomy [12, 16]. Currently, tears in medial discoid menisci are treated with saucerization and suture when the type of lesion allows so [15]. Partial meniscectomy and suture of a torn medial meniscus are more conservative than subtotal meniscectomy but it has not shown better results in the midterm, being subtotal meniscectomy an appropriate choice of treatment for unrepairable tears in a medial discoid meniscus. Results depend on the age at the time of surgery, being worse in children over ten years [17].

Postoperatively the knees were immobilized with a knee orthotic in extension during two weeks allowing partial weight bearing with flexion of the knee limited to 90° the following two weeks. At three months sport was gradually resumed. A generalized rehabilitation protocol has not been established for these kinds of lesions. In sutured bucket handle tears flexion of the knee is from 0° to 90° while bearing weight increases compressive and shear loads in the posterior horn of the meniscus by a factor of 4 [18]. With flexion of the knee the meniscus is displaced posteriorly. The amount of displacement of the meniscus depends on the flexion angle but also on the weight bearing condition [19].

In the case presented the patient had an excellent result, without symptoms at two-year follow-up with the same preinjury level although the patient was 13 years old at the time of surgery.

In the MRI images done two years after the surgery for follow-up there was a reduction of menisci size and increased intensity in T2 signal. We consider that this changes could be caused by radiofrequency saucerization. Wasser et al. reported that, in 6 of the 20 symptomatic discoid meniscus they treated, on the postoperative MRI they found a high signal intensity in T2-weight signal. They considered this finding to be related to the healing process of the sutured discoid meniscus although there was no relation between neither the hypersignal and the surgery performed nor between hypersignal and clinical results [15].

## 4. Conclusion

There is an increasing amount of literature supporting meniscus repair as the treatment of discoid menisci tears in red zone although more long-term follow-up studies need to be done to get better evidence about this subject. The development of different surgical techniques and surgical devices has facilitated this procedure. Suture and saucerization of torn discoid menisci have yielded excellent clinical results, even in meniscal tears difficult to repair, when properly indicated [20]. To the best of our knowledge this is the first case published of bilateral medial discoid menisci tear treated with arthroscopic repair and saucerization in the same patient.

## Figures and Tables

**Figure 8:**